# Molecular evolution of versatile derivatives from a GFP-like protein in the marine copepod *Chiridius poppei*

**DOI:** 10.1371/journal.pone.0181186

**Published:** 2017-07-11

**Authors:** Akihisa Shimizu, Ikuo Shiratori, Katsunori Horii, Iwao Waga

**Affiliations:** Innovation laboratories, NEC Solution Innovators, Ltd., Koto-ku, Tokyo, Japan; Universita degli Studi di Milano-Bicocca, ITALY

## Abstract

Fluorescent proteins are now indispensable tools in molecular research. They have also been adapted for a wide variety of uses in cases involving creative applications, including textiles, aquarium fish, and ornamental plants. Our colleagues have previously cloned a yellow GFP-like protein derived from the marine copepod *Chiridius poppei* (YGFP), and moreover, succeeded in generating transgenic flowers with clearly visible fluorescence, without the need for high-sensitivity imaging equipment. However, due to the low Stokes shift of YGFP (10 nm), it is difficult to separate emitted light of a labeled object from the light used for excitation; hence, limitations for various applications remain. In this study, which was aimed at developing YGFP mutants with increased Stokes shifts, we conducted stepwise molecular evolution experiments on YGFP by screening random mutations at three key amino acids, based on their fluorescent characteristics and structural stabilities, followed by optimization of their fluorescence output by DNA shuffling of the entire coding sequence. We successfully identified an eYGFPuv that had an excitation maximum in UV wavelengths and a 24-fold increase in fluorescence intensity compared to the previously reported YGFP mutant (H52D). In addition, eYGFPuv exhibited almost 9-fold higher fluorescence intensity compared to the commercially available GFPuv when expressed in human colon carcinoma HCT116 cells and without any differences in cytotoxicity. Thus, this novel mutant with the desirable characteristics of bright fluorescence, long Stokes shift, and low cytotoxity, may be particularly well suited to a variety of molecular and biological applications.

## Introduction

Fluorescent proteins are useful visualization markers for studying gene regulation and protein localization, and for live imaging of intermolecular interactions [1–3]. GFP was first identified in the luminescent jellyfish *Aequorea victoria* by Osamu Shimomura (to whom the Nobel Prize was awarded in 2008), and numerous fluorescent proteins with a variety of colors and fluorescence characteristics have been reported to date [4–10]. Recently, there have also been reports of new technologies, including Nano-lantern proteins capable of bioluminescence by fluorescence resonance energy transfer (FRET) between chemiluminescent proteins and fluorescent proteins [11]. Furthermore, the utility of fluorescent proteins has spread widely, not only for research but also for a variety of creative applications, ranging from novelty aquarium fish (https://www.glofish.com/) [12–13] to fluorescent garments made from fluorescent protein-expressing silkworms [14].

Our colleagues previously cloned a yellow GFP-like protein derived from a marine copepod, *Chiridius poppei* (YGFP); this protein shows extremely bright fluorescence, with excitation and emission maxima at 508 nm and 518 nm, respectively [15]. In addition, Sasaki *et al*. has recently succeeded in generating a transgenic *Torenia* flower expressing YGFP, in which the fluorescence was clearly visible at the whole-plant level under excitation light without the highly sensitive imaging equipment normally required [16]. On the other hand, a low Stokes shift is a major drawback of YGFP. In general, fluorophores with long Stokes shifts will be much easier to use in imaging applications than fluorophores with low Stokes shifts, since it is difficult to separate emitted light of a labeled object from the light used for excitation, and further problems involving background fluorescence are often encountered [17]. For reference, Suto *et al*. reported that histidine 52 (H52) is critical to the fluorescent characteristics of YGFP, and that an H52D mutant showed a shorter-wavelength spectrum, albeit with an extremely low fluorescence intensity [18].

In this study, to develop more versatile mutants of YGFP with long Stokes shifts, we first synthesized a mutant DNA library of YGFP in which three amino acids, H52, S133, and R154, were replaced by random sequences. We speculated these three amino acid substitutions might affect the fluorescent characteristics of YGFP, since S133 and R154 are involved in reinforcement of the YGFP chromophore via H52 in the x-ray crystal structure (Protein Data Bank code 2DD7). We expressed this library in *E*. *coli*, and screened for fluorescent YGFP variants by visual inspection of colonies under UV light. Next, we performed DNA shuffling in order to increase the fluorescence intensity of YGFP variants by insertion of random mutations [19]. As a result, we successfully obtained a novel YGFP mutant, eYGFPuv, with excitation maximum blue-shifted to the UV spectrum (400 nm) and with a 24-fold increase in brightness compared to H52D. We also confirmed that eYGFPuv formed dimers in solution, just like the parent YGFP, and exhibited high fluorescence intensity in the acidic pH range (pKa = 3.0). Finally, we revealed that eYGFPuv exhibited an approximately 9-fold increase in fluorescence intensity compared to the commercially available GFPuv [20–21] when expressed in human colon carcinoma HCT116 cells and without any differences in cytotoxicity. These results suggested that this novel mutant with bright fluorescence, long Stokes shift, and low cytotoxicity might be particularly well suited to a variety of molecular and biological applications.

## Materials and methods

### Reagents

Unless otherwise mentioned, all enzymes were purchased form Takara Bio Inc. (Shiga, Japan).

### Generation of mutant YGFP library

A cDNA library encoding YGFP ORF with substitutions at H52, S133, and R154 using random sequences was synthesized and cloned into the pEGFP vector (Clontech, Mountain View, CA, USA) at the *Hind* III and *EcoR* I sites flanking the EGFP sequence. The repertoire of this library was approximately 2.6 × 10^5^. The library constructs described above were synthesized by GenScript (Nanjing, P. R. China).

### Screening of the mutant YGFP library (1^st^ screening)

A visual screen was performed under a 375 nm NS375LIM UV light box (Nitride semiconductors Co., Ltd, Tokushima, Japan). The irradiation aperture was covered with a UL360 filter (OMG Co. Ltd, Osaka, Japan) to eliminate visible light. After transformation and plating of *E*. *Coli* (DH5α cells (Takara Bio Inc), LB plates (approximately 3000 colonies per 100 mm dish) were placed under the UV light box, and visually bright colonies were selected. We selected a total of 100 bright colonies from a total of approximately 3 × 10^5^ colonies and sequenced their YGFP genes using an ABI PRISM 310 Genetic Analyzer (Applied Biosystems, Foster City, CA, USA).

### DNA shuffling (2^nd^ screening)

We introduced random mutations into YGFPuv and YGFPdp sequences, which are both YGFP derivatives obtained from the 1^st^ screening, using a previously described method with certain modifications [20]. The YGFP variant genes were PCR amplified using *ExTaq* (Takara Bio Inc) according to the manufacturer’s instructions, with primers 5ʹ-TTGAATTCATGACAACCTTCAAAATCGAG and 5ʹ-AATTAA-GCTTCTACATGTCTCTTGGGGCGC. The purified PCR product (1.9 μg) was digested into small fragments using DNase I for 20 min at 25°C. DNA fragments between 50 and 300 bp were separated on a 2% agarose gel, and subsequently mixed for PCR at 10–30 ng/μl DNA concentration. The mixed DNA fragments were then diluted 50-fold in a new PCR mixture and re-amplified with the primers described above. The PCR products were cloned into the pEGFP vector employing the *Hind* III and *EcoR* I sites as described above and transformed into *E*. *coli* JM109 cells (Takara Bio Inc). For each cycle of DNA shuffling, approximately 30,000 colonies were obtained. The brightest 20–40 colonies were selected and pooled for the next cycle of visual screening under a UV light box, as described above. We conducted 3 cycles of this DNA shuffling procedure and sequenced the selected YGFP mutants’ genes, as described above.

### Expression and purification of YGFP derivatives and other GFP proteins in *E*. *coli*

We synthesized N-terminal His-tagged YGFP derivatives, GFPuv and EGFP by standard PCR techniques and cloned them into the pEGFP vector at the *Hind* III and *EcoR* I sites, as described above. Protein expression was analyzed in DH5α cells at 37°C without any induction. After overnight incubation, cells were resuspended in PBS containing cOmplete^™^ Mini EDTA-free protease-inhibitor cocktail (Roche, Basel, Switzerland) and sonicated for 3 min (30 sec x 6 pulses) on ice with Q500 (Qsonica, LLC. Newton, CT, US). The soluble protein fraction was mixed with TALON Superflow Metal Affinity Resin (Takara Bio Inc) and incubated overnight at 4°C. After incubation, the resin was washed 5 times with PBS and then washed in PBS containing 200 mM imidazole to elute His-tagged proteins. Eluted proteins were subjected to VIVASPIN4 (Sartorius Stedim, Göttingen, Germany) centrifugal concentration for buffer exchange. To determine protein concentration, purified proteins dissolved in PBS were diluted 2-fold with 10% SDS and then incubated at 95°C for 10 min. Protein concentration of the quenched sample was determined using a BCA protein assay kit (Pierce Biotechnology, Rockland, IL, USA).

### SDS-PAGE and native PAGE analysis

To confirm protein molecular weight and purity, 2 μg of each fluorescent protein (FP) was separated under reducing conditions on a 5–20% gradient polyacrylamide gel (e-PAGEL C520L, ATTO, Tokyo, Japan) in a running buffer containing 0.1% SDS, 25 mM Tris base and 192 mM glycine. To verify the polymerization state of these FPs, 200 pmol of FPs were separated under non-reducing conditions on 10% polyacrylamide gels (c-PAGEL C10L, ATTO) in a running buffer lacking SDS. All gels were stained with GelCode Blue Safe Stain (Thermo Fisher Scientific, Waltham, MA, USA).

### Size exclusion chromatography of YGFP derivatives and other GFP proteins

Hundred microliters of each FP were prepared in 0.5 mg/ml and applied to Superdex 200 Increase 10/300 GL columns (GE healthcare, Chicago, IL, USA) using PBS (20 mM, pH 7.4) at a flow rate of 0.8 ml/min. Protein elution was monitored by measuring optical absorbance at 280 nm (using an ÄKTA Purifier HPLC system, GE Healthcare). To determine the apparent molecular weight, Gel Filtration Calibration Kit LMW (GE Healthcare) was used.

### Absorption spectroscopy and fluorescence spectroscopy

Absorbance spectra of each FP dissolved in PBS was measured with a NanoDrop 2000 instrument (Thermo Fisher Scientific) and fluorescence excitation and emission measurements were performed using a M1000 Pro microplate reader (TECAN, Männedorf, Switzerland). Extinction coefficients of FPs were calculated according to the Beer Lambert law, A = ε*l*c, where “A” is absorption at the excitation maximum, “ε” is the molar extinction coefficient of a certain species, “l” is the path-length, and “c” is the concentration of that given sample. The quantum yields (QY) of each FP were determined according to the formula: QY_unk_ = QY_EGFP_*(A_EGFP_/A_unk)_*(F_unk_ /F_EGFP_) where “A” is absorption of the unknown and EGFP at the excitation maximum, and “F” is the area of the respective emission, which was calculated by using a|e 2.2- UV-Vis-IR Spectral Software (FluorTools, www.fluortools.com). We used the reported EGFP QY of 0.60 [3] as a reference for calculating the quantum yields of YGFP, YGFP variants, and GFPuv. Brightness of each FP was the product of the EC and QY.

### Fluorescence characterization of YGFP derivatives

For pH titrations, 20 μM of each purified FP in PBS (20 mM, pH 7.4) was diluted 10-fold with the following buffers: 0.1 M glycine buffer (pH 3.0–3.5), 0.1 M acetate buffer (pH 4.0–5.5), 0.1 M Phosphate buffer (pH 6.0), 0.1 M HEPES buffer (pH 7.0), 0.1 M Tris-HCl buffer (pH 8.0–9.0), and 0.1 M carbonate buffer (pH 10.0–11.0). In total, 50 μl of FP preparations was incubated at 25°C for 10–80 min, and then added to a 384-well black flat bottom plate and scanned with a M1000 Pro plate reader. For pKa calculations, normalized fluorescence at peak excitation and emission wavelengths were fitted by a standard Henderson-Hasselbalch equation using sma4 for Windows software (Vector Inc. Tokyo, Japan). To determine the thermostability of each protein, 2 μM of each FP in PBS was incubated in a Nexus thermal cycler (Eppendorf, Hamburg, Germany) over a range of 20–80°C for 10 min, then cooled on ice for 5 min.

### Expression of YGFP derivatives and other GFP proteins in human tumor cell lines

N-terminal His-tagged YGFP derivatives, GFPuv and EGFP described above, were cloned into pBApo-EF1α Pur vector (Takara Bio Inc). Expression vectors for transfection were purified using QIAGEN plasmid midi kit (QIAGEN, Venlo, Netherlands). Human colon carcinoma HCT116 cells [22] were purchased from DS Pharma Biomedical (Osaka, Japan) and maintained in McCoy’s 5A medium supplemented with 10% fetal bovine serum. The plasmids were transfected into HCT116 cells using Lipofectamine 2000 (Invitrogen) according to the manufacturer’s instructions. Transfectants were selected using 1μg/ml puromycin (Invitrogen, CA, USA) for 10 days. Fluorescence-positive cells were then sorted using a FACSaria III cell sorter (BD Biosciences, San Jose, CA, USA); the bulk of the sorted cells were used for further analyses. Human breast adenocarcinoma MCF7 cells [23] were maintained in RPMI 1640 medium supplemented with 10% fetal bovine serum; transfection and selection of MCF7 cells were carried out as described for HTC116 cells.

### Flow cytometry

Flow cytometry was carried out on a FACSaria III cell sorter equipped with a 488 nm blue or 405 nm violet laser. Fluorescence readings were recorded using a 502 nm long-pass (LP) mirror with a 530/30 nm band pass filter. Results were analyzed using FlowJo software (Tree Star, Ashland, OR, USA).

### Cytotoxicity measurements

Briefly, 1 × 10^5^ of FACS-purified HCT116- or MCF7 transfectants in 5 ml of growth medium were plated in 6-well plates. After 24 h, the medium was refreshed and incubation continued for an additional 24 h. Harvested cells were serially diluted in fresh medium from 100,000 cells/ ml to 12,500 cells/ ml and we measured the relative number of dead cells using CytoTox-Glo Cytotoxity assay kit (Promega, Madison, WI, USA) according to manufacturer's instructions.

### Real-time PCR analysis

Total RNA of FACS-purified HCT116 transfectants was prepared using RNeasy Mini kits (Qiagen). RT-qPCR analysis was performed using PrimeScript High Fidelity RT-PCR kit (Takara Bio Inc) with SYBYR Gold (Thermo Fisher Scientific) and a CFX96 Real-Time system instrument (Bio-Rad, Hercules, CA, USA).

## Results

### Expression cloning of YGFPdp, and YGFPuv from YGFP mutant library

**Fig 1:**
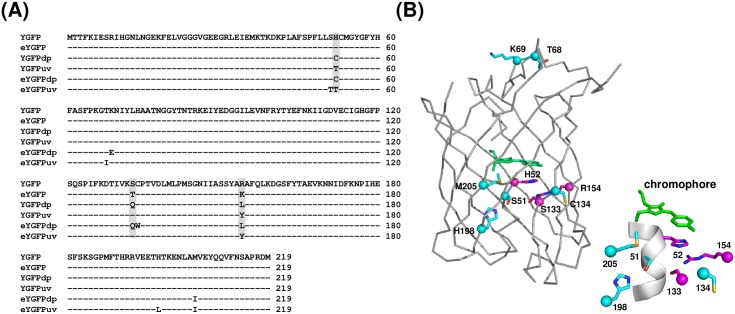
Amino acid sequence alignment of YGFP and its variants identified in this study. (A) Shaded residues were replaced with random amino acids for the 1st library screening. (B) Overall structure of CpYGFP (Accession No. AB185173 and Protein Data Bank code 2DD7). The structure model was drawn using PYMOL software (DeLano Scientific; http://www.pymol.org). Amino acid mutations of eYGFPdp and eYGFPuv, both obtained from DNA shuffling, are shown as cyan spheres with side chains. The chromophore composed of a tripeptide, Gly-Tyr-Gly, in the center is shown in the stick model (green). Magenta spheres indicate the amino acids at positions 52, 133, and 154, which are described in (A). The sequences of these YGFP variants in this figure are available at the DDBJ/EMBL/GenBank nucleotide sequence databases with the accession numbers LC217529 (eYGFP), LC17530 (YGFPdp), LC217531 (YGFPuv), LC217532 (eYGFPdp) and LC217533 (eYGFPuv), respectively.

**Fig 2:**
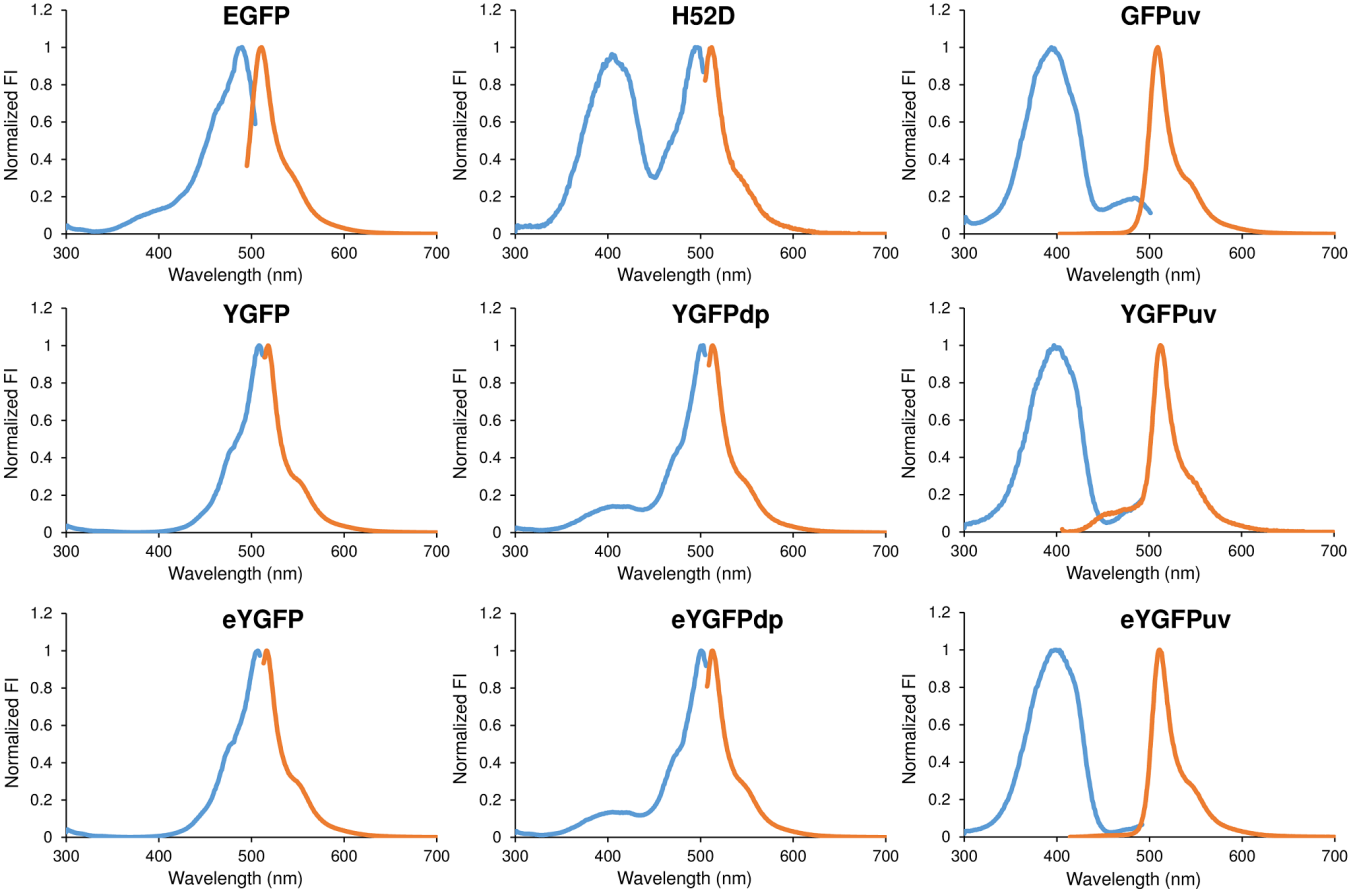
Fluorescence properties of YGFP and its variants in comparison to EGFP and GFPuv. Fluorescence excitation and emission spectra of YGFP, YGFP derivatives and other GFP proteins. Purified FPs were diluted to 2 μM in PBS (20 mM, pH 7.4) and scanned by TECAN M1000 Pro hardware. Normalized excitation (blue line) and emission (orange line) spectra are shown with emission and excitation maxima described in Table 1, respectively. All experiments in this figure were repeated three times with each data point measured in triplicate; representative data are shown.

Based on the structural analysis of wild-type YGFP by Suto *et al*., loss of the π-π stacking interaction between the internal tripeptide chromophore (Gly55-Tyr56-Gly57) and the H52 side chain is responsible for changing both excitation and emission wavelengths [18]. This report also showed that the excitation spectrum of H52D mutant was blue-shifted. However, the excitation spectrum shift was partial, and the fluorescence intensity was extremely low. To develop a mutant with an excitation maximum within ultra-violet wavelengths and with a strong fluorescence emission, we identified two additional amino acid positions that might contribute to the fluorescent characteristics of YGFP. One of these is S133 and the other is R154. Since S133 and R154 directly interact with H52 and each other, both amino acids are likely to affect the chromophore via H52 if replaced. Next, we synthesized a mutant library of YGFP to be expressed in *E*. *coli*, whereby the three amino acid positions described above were replaced with random sequences. In the 1^st^ screening, we selected 100 colonies from approximately 3 × 10^5^ colonies exhibiting significant fluorescence under UV illumination, and analyzed their YGFP gene sequences. From this initial screen, we identified two mutants, one was YGFPdp (dp indicates “dual peak”), and the other was YGFPuv. The specific mutations of these YGFP mutants are outlined in Fig 1. Subsequently, we prepared purified proteins with a His-tag added to the N-terminus and conducted scans of their fluorescence spectra over a broad wavelength range. The normalized excitation and emission spectra of these YGFP mutants dissolved in PBS (20 mM, pH 7.4) are shown in Fig 2, and fluorescence properties are summarized in Table 1. For reference, the extinction coefficient (EC) of YGFP calculated in our laboratory was 101,000 M^-1^cm^-1^, which is comparable to the published value of 95,000 M^-1^cm^-1^ [15]. The excitation spectrum of YGFPdp was partially blue-shifted similar to that observed for the H52D substitution, and exhibited a major peak at 502 nm and a minor peak at 409 nm. Notably, the excitation spectrum of YGFPuv was fully blue-shifted (398 nm). EC and brightness of YGFPuv were approximately 7-fold higher than that of H52D.

**Table 1:** Spectral properties of YGFP and its variants in comparison to EGFP and GFPuv.

	Ex (nm)	Em (nm)	EC (M^-1^cm^-1^)	QY	Brightness (EC*QY)	Theoretical MW (kDa)	Calculated MW (kDa)	Oligomeric states	Ref.
EGFP	488	511	53,300	0.60	32,000	28.1	31.1	Monomer	This work
GFPuv	394	509	27,800	0.80	22,200	28.0	34.6	Monomer	This work
YGFP	508	518	101,000	0.53	53,500	25.9	46.6	Dimer	This work
H52D	497 (411)	512	1,200	0.80	1,000	25.9	N.D.	Dimer	This work^a^
eYGFP	507	516	124,000	0.58	71,900	25.9	N.D.	Dimer	This work
YGFPdp	502 (409)	513	17,300	0.70	12,100	25.9	44.6	Dimer	This work^a^
YGFPuv	398	512	8,800	0.81	7,100	25.9	N.D.	Dimer	This work
eYGFPdp	501 (405)	513	49,800	0.69	34,300	25.9	48.9	Dimer	This work^a^
eYGFPuv	398	512	31,200	0.76	23,700	25.9	44.8	Dimer	This work

The excitation (Ex) and emission (Em) wavelengths, molar extinction coefficient (EC), quantum yield (QY), brightness, theoretical molecular weight (MW), calculated MW estimated by size exclusion chromatography, and oligomeric states estimated by native PAGE and size exclusion chromatography are listed. All values in this table were calculated in this study except for the reported QY of EGFP [3], which was used as a reference for calculating the quantum yield of YGFP variants and GFPuv. For reference, reported ECs of EGFP, GFPuv and YGFP were 56,000 M^-1^cm^-1^ [3], 30,500 M^-1^cm^-1^ [21] and 95,000 M^-1^cm^-1^ [15], and reported QY of GFPuv was 0.79 [21], respectively.

^a^ EC, QY and brightness were determined by using absorbance at the major peak of excitation spectrum. N.D.: not determined.

### Expression and cloning of eYGFPdp and eYGFPuv by DNA shuffling

Next, we investigated whether the fluorescence intensity of YGFPdp and YGFPuv could be further enhanced. We referred to the report by Crameri *et al*. and performed DNA shuffling using the YGFPdp and YGFPuv sequences as templates [20]. After three cycles of shuffling, we identified enhanced YGFPdp (eYGFPdp) and enhanced YGFPuv (eYGFPuv_)_ (Fig 1). The excitation spectrum of purified eYGFPdp exhibited a major peak at 501 nm and a minor peak at 405 nm, while its EC and brightness at peak excitation wavelength (501 nm) were approximately three-fold higher than those of YGFPdp (Fig 2 and Table 1). The EC and brightness of eYGFPuv were approximately four- and three-fold higher than that of YGFPuv and nearly 1/3^th^ and half the intensity of wild-type YGFP, respectively.

### Expression cloning of eYGFP from YGFP mutant library

In addition to YGFPdp and YGFPuv, an eYGFP was also obtained in the 1st screening. eYGFP showed almost the same fluorescence spectrum as wild-type YGFP. Since the mutations in the mutant were conservative in nature, S133 to T and R154 to K, it is not surprising that no significant changes were observed. However, EC, QY and brightness of eYGFP were clearly higher than those of YGFP (Table 1). Furthermore, FACS analysis revealed that the average fluorescence intensity of *E*. *coli* cells expressing eYGFP excited by a 488 nm blue laser was 3-fold higher than cells expressing wild-type YGFP; however, this is due to higher expression levels of eYGFP and not due to any intrinsic properties of eYGFP (S1 Fig).

### Comparative spectral analysis of YGFP, YGFP derivatives, EGFP and GFPuv

Fluorescence properties of commercially available EGFP and GFPuv, which are used in many types of research, were compared with those of YGFP derivatives (Fig 2 and Table 1). For reference, EC of YGFP and GFPuv calculated in our laboratory were 53,300 M^-1^cm^-1^ and 27,800 M^-1^cm^-1^, respectively; both ECs were slightly lower than the published values of 56,000 M^-1^cm^-1^ [3] and 30,500 M^-1^cm^-1^ [21]. On the other hand, the QY of GFPuv calculated in our laboratory was 8.0, which was almost identical to the published value of 0.79 [21]. The EC and brightness of the eYGFP were approximately 2-fold higher than those of EGFP, and the EC and brightness of eYGFPuv were slightly higher than those of GFPuv. We also visually compared the fluorescence strength of YGFP and its derivatives with those of EGFP and GFPuv by taking photographs under the same imaging conditions (S2 Fig). In correlation with the results of fluorescence spectroscopy, we could observe the bright fluorescence of eYGFPuv as well as that of GFPuv when excited by a hand-held UV LED light (385 nm) without any filter, while we could observe the strongest fluorescence of eYGFP excited by a blue LED light (470 nm) through a yellow-colored optical filter. Overall, by conducting stepwise screening, we successfully identified not only improved YGFP (eYGFP), a by-product of mutant library screening, but also eYGFPuv exhibiting excitation maxima in UV wavelengths with a longer Stokes shift and a brightness 24-fold higher than that of the previously reported H52D mutant.

### Oligomeric states of YGFP mutants

**Fig 3:**
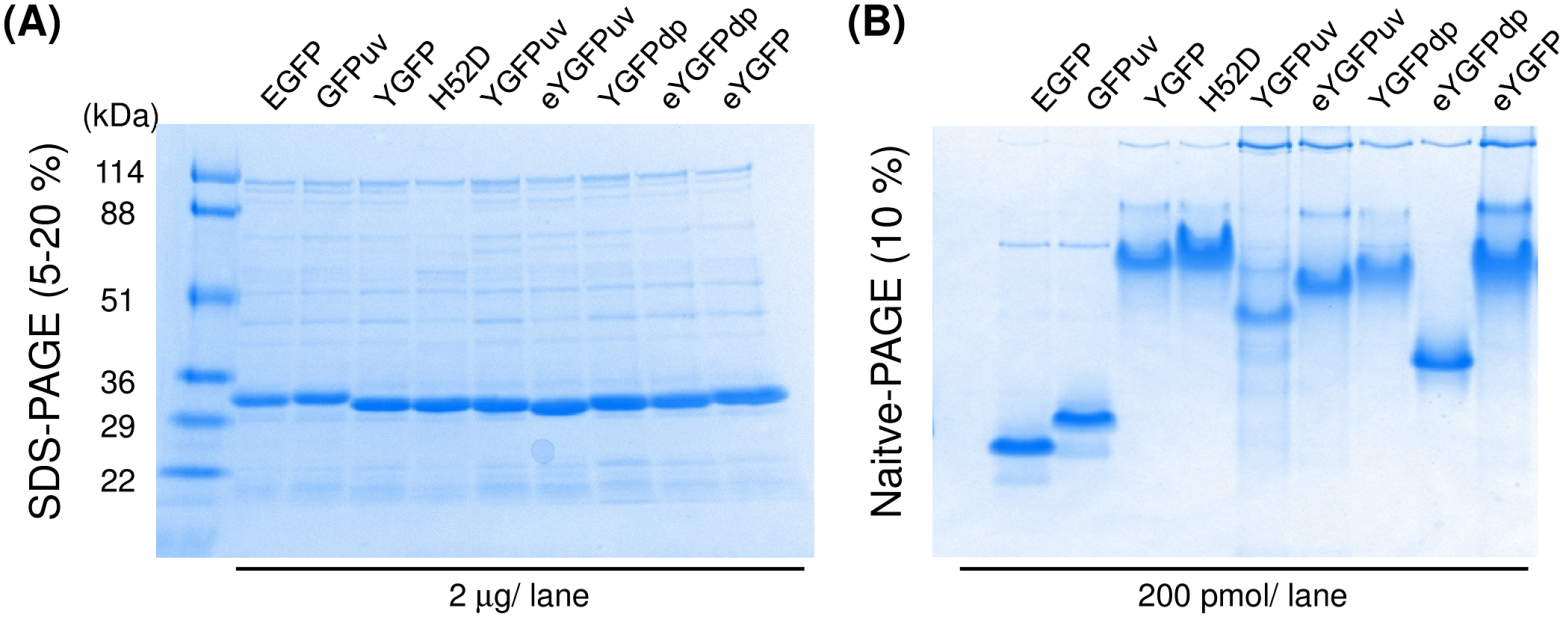
SDS-PAGE and native PAGE analysis of YGFP and its variants in comparison to EGFP and GFPuv. (A) N-terminal His-tagged proteins were separated by SDS-PAGE in a 5–20% polyacrylamide gel under reducing conditions. The gel was stained with Coomassie blue. (B) Oligomeric states of YGFP derivatives or commercial GFP proteins were visualized after 10% non-denaturing gel electrophoresis. Data are representative of two independent experiments.

The oligomeric state is an important parameter for the application of FPs as fusion tags [24]. Masuda *et al*. reported that YGFP forms dimers [15]. However, this previous report also showed that aggregated particles of YGFP were rarely seen in transfected HeLa cells, and YGFP-tagged actin appeared to be properly assembled into characteristic actin filaments, judging from microscopic analysis. To determine the oligomeric states of YGFP mutants, we first conducted native PAGE analysis and confirmed that the mass of YGFP and its derivatives were all greater than that of EGFP and GFPuv, which are known to be monomers at low protein concentrations (Fig 3) [21, 25]. Next, we calculated the apparent molecular weight of YGFP variants by size exclusion chromatography, using a gel-filtration column equilibrated with PBS. The apparent molecular weight of YGFP and the derivative variants tested showed approximately 1.7–1.8 times higher molecular mass than the theoretical values predicted from their respective amino acid sequences (Table 1 and S3 Fig). On the other hand, the apparent molecular weight of EGFP and GFPuv were only slightly greater than the theoretical molecular weight. Judging from these results, we speculated that YGFP derivatives as well as intact YGFP formed dimers under physiological conditions.

### pH-dependence of YGFP mutants

**Fig 4:**
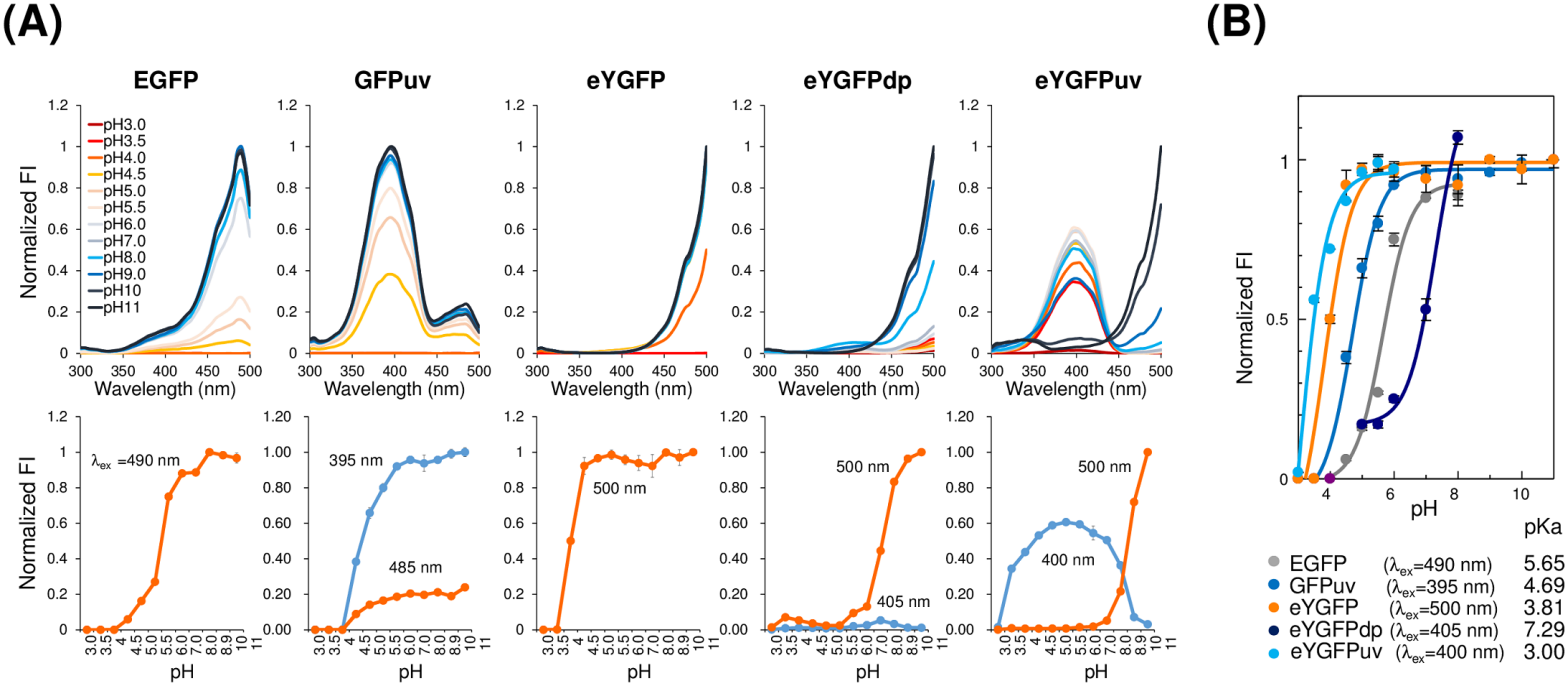
pH-dependence of YGFP and its variants in comparison to EGFP and GFPuv. (A) Normalized pH-dependent excitation spectra at peak emission wavelengths (upper panel) and plot of fluorescence versus pH at indicated excitation wavelengths and peak emission wavelengths (lower panel). In total, 20 μM of each fluorescent protein in PBS (20 mM, pH = 7.4) were diluted 10-fold with the indicated pH solutions and incubated at 25°C for 80 min. (B) Normalized fluorescence at indicated excitation wavelengths were fitted by a standard Henderson-Hasselbalch equation for determination of their pKa values. All experiments in this figure were repeated three times with each data point measured in triplicate; representative data are shown.

In general, most fluorescent proteins are stable between pH6-10, but at pH < 6, stability is decreased with some acid sensitivity [1–2, 26]. To investigate pH-dependence of YGFP derivatives, we initially determined the incubation period required for fluorescence of eYGFPuv to reach equilibrium and confirmed that 80 min incubation was necessary for stabilizing the fluorescent properties of eYGFPuv (S4 Fig). The pH-dependent excitation spectral changes of EGFP, GFPuv, eYGFP, eYGFPdp, and eYGFPuv are shown in Fig 4A and the results of pKa calculations for each FP are presented in Fig 4B. pKa of EGFP and GFPuv, as calculated in our laboratory, were 5.65 and 4.69, respectively; both values are almost coincident with previously reported values of 5.65 (EGFP) [27] and 5.0 (GFPuv) [28]. Interestingly, fluorescence properties of eYGFPuv under alkaline conditions (pH = 10.0–11.0) were completely reversed to those of YGFP and, as a result, fluorescent intensity at an excitation wavelength of 400 nm was markedly decreased. Because of this fluorescence change of eYGFPuv, pKa of eYGFPuv could be calculated only if the fluorescence plot was fitted among a limited pH range of 3.0–6.0. Notably, eYGFPuv exhibited marked fluorescence intensity under acidic conditions (pKa = 3.0) in comparison to EGFP and GFPuv. eYGFP was also less prone to be affected by acidic conditions (pKa = 3.8) compared to EGFP and GFPuv. eYGFPdp also exhibited marked change in excitation spectrum in the same manner as eYGFPuv, and in contrast to the other FPs tested, a pKa of 7.29, which could be calculated only if the fluorescence plot was fitted among a limited pH range of 5.0–8.0, suggesting that eYGFPdp might be unstable even under neutral conditions.

### Chemical stability and thermostability of YGFP derivatives

**Fig 5:**
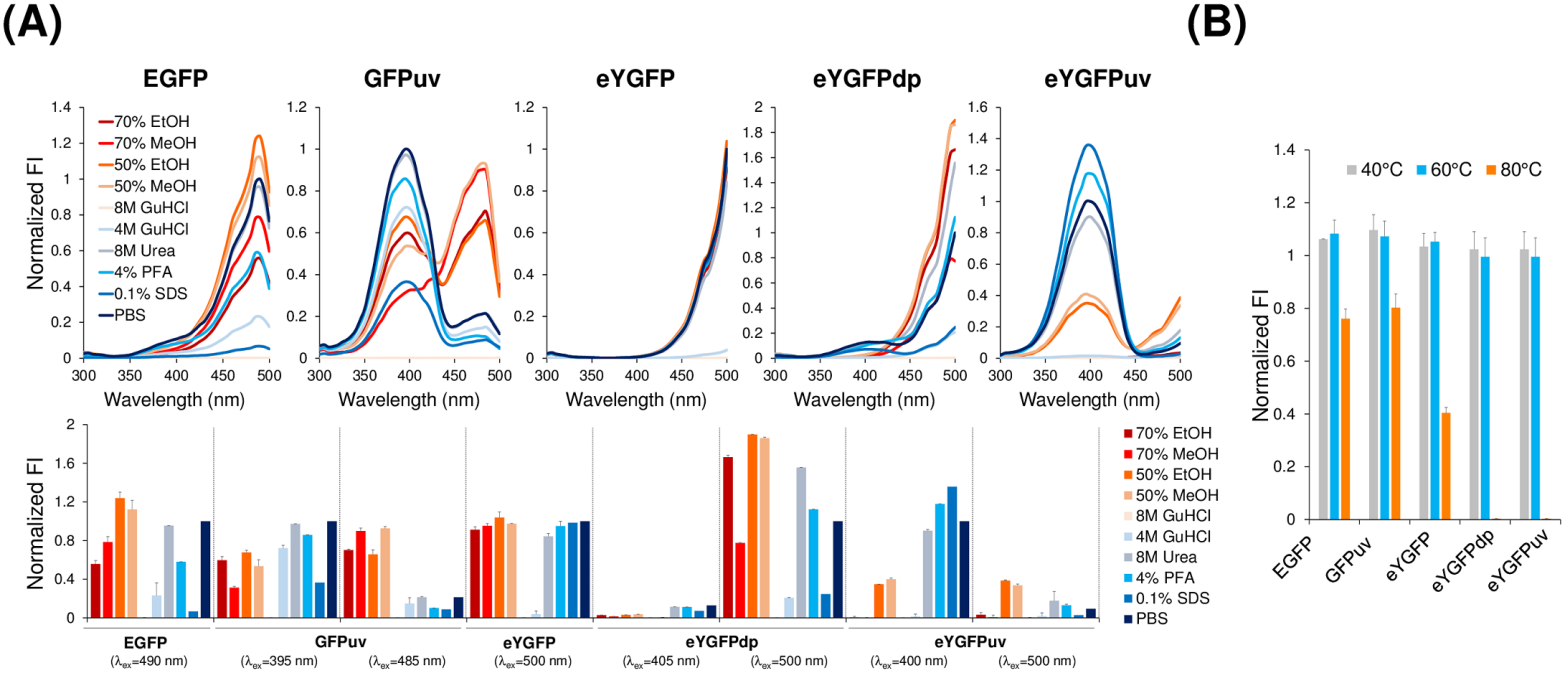
Chemical stability and thermostability of YGFP and its variants in comparison to EGFP and GFPuv. (A) 20 μM of each fluorescent protein in PBS was diluted 10-fold with the indicated reagents and incubated at 25°C for 80 min. Fluorescent excitation spectra at peak emission wavelength (upper panel) and fluorescence at indicated excitation wavelengths and peak emission wavelengths (lower panel) were normalized to a control sample diluted in PBS. (B) Thermostability of fluorescent proteins. In total, 2 μM of each fluorescent protein in PBS was heated at the indicated temperature for 10 min and fluorescence at the peak excitation and emission wavelengths were measured at 25°C. All experiments in this figure were repeated three times with each data point measured in triplicate; representative data are shown.

To compare the effects of solvents and chaotropic agents on the fluorescent properties of YGFP derivatives under similar conditions to those described in a previous report [15], we initially determined the incubation period required for the fluorescence of eYGFPuv to reach equilibrium and confirmed that an 80 min incubation was necessary for stabilizing the fluorescence properties of eYGFPuv, as is the case with the pH-dependence described in Fig 4 (S5 Fig). Chemical stability of EGFP, GFPuv, eYGFP, eYGFPdp and eYGFPuv are shown in Fig 5A, and the results of thermostability assays for each FP are presented in Fig 5B. Notably, eYGFP exhibited relatively high tolerance to many solvents and chaotropic agents tested except in the case of incubation in guanidium chloride (Gu-HCl). eYGFPuv as well as eYGFP were more resistant to incubation in 0.1% SDS compared to the other FPs tested. On the contrary, YGFP derivatives were more sensitive to Gu-HCl compared to EGFP and GFPuv. Interestingly, the excitation spectra of GFPuv treated with alcohols were partially red-shifted and the excitation spectrum of eYGFPdp was also completely reversed to that of YGFP. Furthermore, we found that eYGFPuv was markedly influenced by the inclusion of alcohol among the FPs tested. However, the reduction in fluorescent intensity of eYGFPuv at an excitation wavelength of 400 nm was also not entirely due to quenching, but to red-shifting of the excitation spectrum to the same excitation wavelength as that of the wild-type. In terms of thermostability, eYGFPdp and eYGFPuv had lower thermostability than did eYGFP, EGFP, and GFPuv (Fig 3B). The relative fluorescent intensity of eYGFPdp and eYGFPuv at 80°C were approximately <1% of their intensities at 25°C, and approximately 100-fold lower than that of eYGFP.

### Expression of YGFP mutants in human tumor cell lines

**Fig 6:**
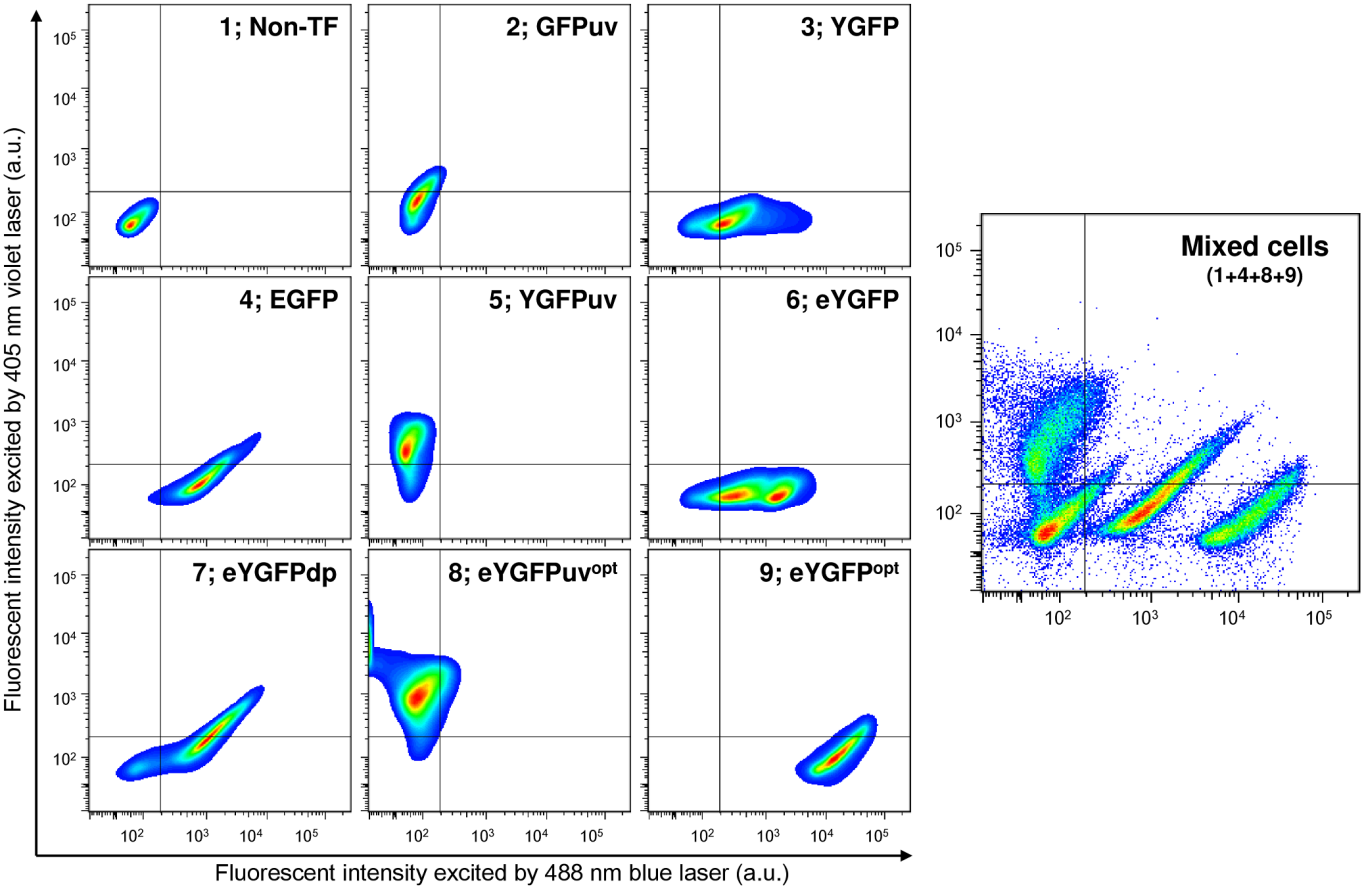
FACS analysis of HCT116 cells expressing YGFP derivatives or commercial GFP proteins. Transfectants (TF) were selected using 1 μg/ml puromycin for 10 days, and fluorescence-positive cells were sorted by FACSaria III, as described in Materials and Methods. eYGFP^opt^ and eYGFPuv^opt^ nucleotide sequences are available from the DDBJ/EMBL/GenBank nucleotide sequence databases with accession numbers LC217534 (eYGFP^opt^) and LC217535 (eYGFPuv^opt^), respectively. Data are representative of three independent experiments.

The extinction coefficient and brightness of purified eYGFPuv were slightly higher than those of GFPuv, and the extinction coefficient and brightness of purified eYGFP were significantly higher than those of wild-type YGFP (Table 1). On the other hand, FACS analysis results demonstrated that the average fluorescence intensity of *E*. *coli* cells expressing eYGFP excited by 405 nm violet laser light was 19-fold higher than that of cells expressing GFPuv. Similarly, the average fluorescence intensity of *E*. *coli* cells expressing eYGFP excited by a 488 nm blue laser was approximately 3-fold higher than that of cells expressing wild-type YGFP and 36-fold higher than that of cells expressing EGFP (S1 Fig). It should be noted that the expression vectors for all GFP proteins, with His-tags fused to the N-termini, were of the same design (see Materials and methods), but codon usage was not optimized for bacterial expression, whereas the sequence of EGFP used in this study is well known to be optimized for mammalian expression [29]. Therefore, to compare the expression levels in mammalian cells, we expressed the same His-tagged fluorescent proteins in HCT116 cells derived from human colon cancer, and sorted the fluorescence-positive cells by FACS. Additionally, we prepared eYGFP^opt^ and eYGFPuv^opt^, which use codons optimized for expression in human cells. FACS analysis results for pre-sorted cells are shown in S6 Fig. Cells expressing eYGFPuv showed slightly higher fluorescence than cells expressing GFPuv in the 405 nm excitation channel, while cells expressing eYGFPuv^opt^ showed significantly higher fluorescence signal than cells expressing eYGFPuv. Cells expressing EGFP showed brighter fluorescence than cells expressing YGFP, eYGFP, and eYGFPdp in the 405 nm channel or 405 nm-488 nm double-positive quadrant, while cells expressing eYGFP^opt^ showed very much stronger fluorescence than cells expressing EGFP, as detected in the 488 nm excitation channel. FACS analysis results of sorted cells are shown in Fig 6 and Table 2. Cells expressing eYGFPuv^opt^ were almost in the 405 nm single quadrant as observed with cells expressing GFPuv. The average fluorescence intensity in the 405 nm channel of cells expressing eYGFPuv^opt^ was 4-fold higher than that of cells expressing eYGFPuv, and 9-fold higher than that of cells expressing GFPuv. Cells expressing eYGFP^opt^ also showed extremely strong fluorescence in the 488 nm single quadrant, as observed with cells expressing YGFP and eYGFP. The average fluorescence intensity in the 488 nm channel of cells expressing eYGFP^opt^ was 19-fold higher than those of cells expressing eYGFP and EGFP. Finally, we analyzed a mixture of cells; non-transfected, EGFP-, eYGFPuv^opt^-, and eYGFP^opt^- transfected. Each population was clearly distinguishable, and again, cells expressing eYGFPuv^opt^ and eYGFP^opt^ showed very much stronger fluorescence than cells expressing EGFP. Similar findings were also observed when MCF7 cells were used as transfection recipients (S7 Fig and S1 Table).

**Table 2:** Normalized fluorescence intensity of HCT116 cells expressing various FPs.

	Normalized mean fluorescence intensity	
Excitation wavelength (nm) length	405 nm	488 nm
EGFP	2.1 ±0.018	16.5 ±0.033
GFPuv	2.9 ±0.020	1.3 ±0.100
YGFP	1.0 ±0.004	6.8 ±0.030
eYGFP	1.1 ±0.007	16.5 ±0.054
eYGFPdp	3.9 ±0.046	20.6 ±0.088
eYGFPuv	7.0 ±0.063	0.8 ±0.148
eYGFPuv^opt^	26.1 ±0.009	1.4 ±0.016
eYGFP^opt^	2.0 ±0.102	307.7 ±0.031

The averages of fluorescence intensities for each fluorescent channel were normalized with those of non-transfected control cells. The values represent the average of three independent experiments.

### Cytotoxicity of YGFP derivatives expressed in in human tumor cell lines

Fluorescent proteins are widely utilized to label cells for tracking. However, some evidence has been reported that transfection/transduction of FPs causes cellular damage by inducing the generation of reactive oxygen species or by initiating apoptosis [30]. Indeed, some FPs are reported to be toxic, and stable transformants with high expression levels are difficult to establish [30]. Therefore, we evaluated cytotoxicity of YGFP derivatives by measuring cell viability of HC116 transfectants, as described in Fig 6. As a result, there were no differences in cell viability among the cells tested (Table 3 and S8 Fig). However, abundance of FP transcripts for each transfectant were relatively well-correlated with fluorescence intensity of each transfectant; thus, the low cytotoxicity of YGFP derivatives might partly contribute to high expression levels in mammalian cells (Table 3).

**Table 3:** Summary of cell viability and FP transcription levels in HCT116 cells expressing various FPs.

	Viability (%)	Normalized transcription levels of FP
Non-transfected	91.1 ±2.9	N.D.
EGFP	91.6 ±2.0	0.14 ±0.003
GFPuv	91.6 ±2.7	0.02 ±0.001
eYGFPuv^opt^	91.8 ±3.1	0.16 ±0.005
eYGFP^opt^	92.4 ±2.51	0.25 ±0.013

Transcription levels of FP were normalized by comparison with those of GAPDH, and the relative amounts of FP transcripts are shown. Primers used for amplification were shown in S2 Table. All experiments in this figure were repeated two times with each data point measured in triplicate; representative data are shown.

## Discussion

Here, we successfully developed a novel YGFP derivative, eYGFPuv, which exhibited an excitation maximum in the UV region (400 nm, Fig 2 and Table 1) and higher fluorescence emission than that of GFPuv *in vitro* (Table 1) and *ex vivo* (Table 2). Wild-type YGFP has even stronger fluorescence emission, but, the maximum excitation is within the visible light spectrum (508 nm), which gives a Stokes shift of only 10 nm from its maximum emission wavelength (518 nm). We successfully achieved an increase in the Stokes shift from 10 nm to 114 nm by conducting stepwise molecular engineering, which involved replacement of only 6 amino acids (Fig 1, Table 1). In general, a long Stokes shift fluorescence in FPs occurs when the pKa of the neutral phenol form of the chromophore substantially decreases upon excitation, leading to excited state proton transfer (ESPT) and emission of fluorescence from the anionic phenolate form [31]. In this regard, His52 forming stacking interaction with the phenol moiety of the YGFP chromophore and Arg154 undergoing stacking interaction with His52 were reported to be involved in altering emission wavelength and stabilization of the chromophore [18]. Because the H52T mutation in eYGFPuv and the H52C substitution in eYGFPdp broke the stacking interaction with the phenol moiety of the chromophore, and the R154 in both eYGFPuv and eYGFPdp was substituted by a hydrophobic amino acid (eYGFPuv with R154Y, eYGFPdp with R154L), we hypothesized that these mutations altered the hydrogen bond network around the chromophore, followed by alteration to the ESPT pathway. Interestingly, we initially hypothesized that both A133 and R154 might each interact with H52 and so both mutations would be likely to affect the chromophore via H52 if replaced. However, because the S133 mutation was observed only in dp mutants (YGFPdp and eYGFPdp) and not in UV mutants (YGFPuv and eYGFPuv), the S133Q substitution in dp mutants was not likely to be critical for the stability of YGFP fluorescence but, rather, compensating for the H52C and R154L replacements.

In both eYGFPuv and eYGFPdp mutants, M (ATG) is substituted to I (ATA) at position 205. Despite the low codon frequency for ATA in *E*. *coli*, this mutation was nonetheless selected during DNA shuffling. From a structural standpoint, in addition to H52, M205 is also presumed to interact with the chromophore, and thus M205I substitution in both mutants may critically affect interactions of this residue with the chromophore, which in turn may modulate the structural stability and folding of the overall YGFP polypeptide. Regarding the H198L mutation in eYGFPuv, while residue 198 of YGFP is relatively distant from the chromophore, the side chain of H198 faces inside YGFP, and thus the H198L mutation may affect chromophore formation by interaction of this residue with the 47−54^th^ alpha helices. In addition, given that the C134W mutation of eYGFPdp and the S51T substitution of eYGFPuv are adjacent to Q133 and T52, respectively, it is conceivable that they also affect the interaction network surrounding the chromophore. Conversely, the K69E mutation of eYGFPdp is distant from the chromophore; the residue at this position is unlikely to interact directly with the chromophore, although mutation at this position is likely to affect protein stability and folding.

Interestingly, eYGFPuv and eYGFP exhibited high emission intensities in the acidic pH range (eYGFPuv; pKa = 3.0, eYGFP; pKa = 3.8), which were unusual compared to other FPs such as EGFP (pKa = 5.7) (Fig 4 and S4 Fig). On the contrary, at high pH (10.0–11.0), the fluorescence properties of eYGFPuv and eYGFPdp were red-shifted and reversed to those of wild-type. From a structural standpoint, we hypothesized this pH-dependent shift of excitation spectra at extremely alkaline pH might be induced by the ionization of Tyr56 in the chromophore and/or by the deprotonation of Thr136 that stabilizes the enolate form of the chromophore imidazolinone (S9 Fig) [32–33]. Nonetheless, because eukaryotic cells contain a variety of defined compartments, not only cytosol (pH = 6.8–7.2) but also acidic vesicles and organelles (pH 4.7–6.5) [34], eYGFPuv and eYGFP might have some advantages for live cell imaging compared with other FPs. Regarding chemical stability, eYGFPuv was markedly influenced by alcohol treatment. Unfortunately, we were unable to explain the quenching mechanism; however, partial red-shifting might occur in the same manner as the pH-dependent shift described above.

While the fluorescence intensity of purified eYGFPuv protein is slightly higher than commercial GFPuv, FACS analysis showed that the fluorescence intensity of *E*. *coli* cells expressing eYGFPuv was 19-fold higher than that of GFPuv-expressing cells (Table 1 and S1 Fig). Similar results were obtained in HCT116 cells; eYGPuv^opt^-expressing cells had 9.2-fold higher fluorescence than GFPuv expressing cells (Fig 6 and Table 2). Furthermore, an extremely strong fluorescence was detected for HCT116 cells expressing eYGFP^opt^, and the fluorescence intensity in the 488 nm channel was up to 18-fold higher than that observed in EGFP expressing cells. In this regard, the viability of eYGFPuv^opt^-and eYGFP^opt^ expressing cells were comparable to other tested cells, and amounts of FP transcript were relatively well-correlated with the fluorescent intensity of each transfectant (Table 3). Therefore, we hypothesized that the low cytotoxicity of YGFP derivatives might partly contribute to high expression levels in *E*. *coli* and mammalian cells, although details of this mechanism remain speculative at this point. Furthermore, because EGFP fluorescence was detected across both channels (blue and UV light excitation), as well as fluorescence of eYGFPdp (Fig 6), the combination of eYGFPuv (or eYGFPuv^opt^) and eYGFP (or eYGFP^opt^), both of which are fluorescent only in their respective channels, can be used more effectively in multi-channel excitation than use of EGFP or eYGFFPdp alone.

In conclusion, although further characterization of these YGFP derivatives as well as evaluation of *in vivo* imaging applications are needed, we believe that these mutants provide insight into the influence of the protein structure on YGFP’s fluorescence and may serve as starting points for future efforts developing various biological applications.

## Supporting information

S1 Fig Flow cytometry of *E*. *coli* DH5α cells expressing various FPs. A His-tag was fused to the N-terminus of each FP. Flow cytometry was carried out using the same settings as described in Fig 5. The average fluorescence intensity of eYGFP expressing cells in the 488 nm channel was 3-fold greater than that of YGFP expressing cells. The mean fluorescence intensity of eYGFPuv expressing cells in the 405 nm channel was 20-fold greater than that of GFPuv expressing cells. The values represent the average of three independent experiments.
(TIFF)

S2 Fig Visual fluorescence comparison of different GFP proteins. Photograph of bacterial colonies of each FP construct at indicated concentrations were taken under ultraviolet LED light (model LED-UV385P, OptoCode, Tokyo, Japan) without filters or blue LED light (model LED470-3WOF, OptoCode) with a yellow optical filter (SC52, Fujifilm, Tokyo, Japan) by using a Canon EOS Kiss Digital X7i camera (Canon, Tokyo, Japan). Image acquisition conditions: F5.6, ISO200, 1 s exposure, focal length 55 mm. Representative data from more than three independent experiments are shown.
(TIFF)

S3 Fig Size exclusion chromatography of YGFP derivatives and other GFP proteins. (A) 50 μg of each FP in PBS (20 mM, pH = 7.4) was loaded onto a Superdex 200 Increase 10/300 GL column. Expected elution volumes are indicated. (B) Standard curve for calibration of S200 size exclusion column.
(TIFF)

S4 Fig pH- and time-dependent fluorescence spectral changes of eYGFPuv. pH- and time-dependent changes of excitation spectra of eYGFPuv with emission maxima at 508 nm (upper panel) and plot of fluorescence versus pH at indicated excitation wavelength and peak emission wavelength (lower panel). In total, 20 μM of each fluorescent protein in PBS was diluted 10-folf with the indicated pH solutions and incubated at 25°C for the specified time durations. All experiments in this figure were repeated three times with each data point measured in triplicate; representative data are shown.
(TIFF)

S5 Fig Time-course analysis for chemical stability of eYGFPuv. Fluorescence excitation spectra of eYGFPuv with emission maxima at 508 nm (upper panel) and fluorescence at indicated excitation wavelength and peak emission wavelength (lower panel) were normalized to a control sample diluted in PBS. In total, 20 μM of each fluorescent protein in PBS was diluted 10-fold with the indicated reagents and incubated at 25°C for the specified time durations. All experiments in this figure were repeated three times with each data point measured in triplicate; representative data are shown.
(TIFF)

S6 Fig FACS analysis of pre-sorted HCT116 cells expressing various FPs. HCT116 cells expressing various FPs were prepared as described in Materials and Methods. FACS analysis was carried out using the same settings as described in Fig 6. Data are representative of two independent experiments.
(TIFF)

S7 Fig FACS analysis of MCF7 cells expressing YGFP derivatives or commercial GFP proteins. Preparation of MCF7 cells expressing various FPs and FACS analysis of sorted cells were as described for HCT116 cells. Mean fluorescence of purified cells is shown in Supplemental Table 1. Data are representative of three independent experiments.
(TIFF)

S8 Fig Cytotoxicity assay of HCT116 and MCF7 cells expressing various FPs. Cells were prepared as described in Materials and Methods. The experiment was repeated three times with each data point measured in triplicate; representative data are shown.
(TIFF)

S9 Fig Speculation for the shift of excitation spectra at extremely alkaline pH. The structure model was drawn using PYMOL software as described in Fig 1.
(TIFF)

S1 Table Summary of mean values of MCF7 cells expressing various fluorescent proteins. The averages of fluorescence intensities for each fluorescent channel were normalized to those of non-transfected cells. The values represent the average of three independent experiments.
(TIFF)

S2 Table Primer sets used for RT-qPCR described in Table 3. (TIFF)
