# Supplementary figures and images for: Molecular evolution of versatile derivatives from a GFP-like protein in the marine copepod Chiridius poppei
Source: PLoS One. 2017 Jul 11;12(7):e0181186. doi: 10.1371/journal.pone.0181186 (PMC5507436; doi:10.1371/journal.pone.0181186)

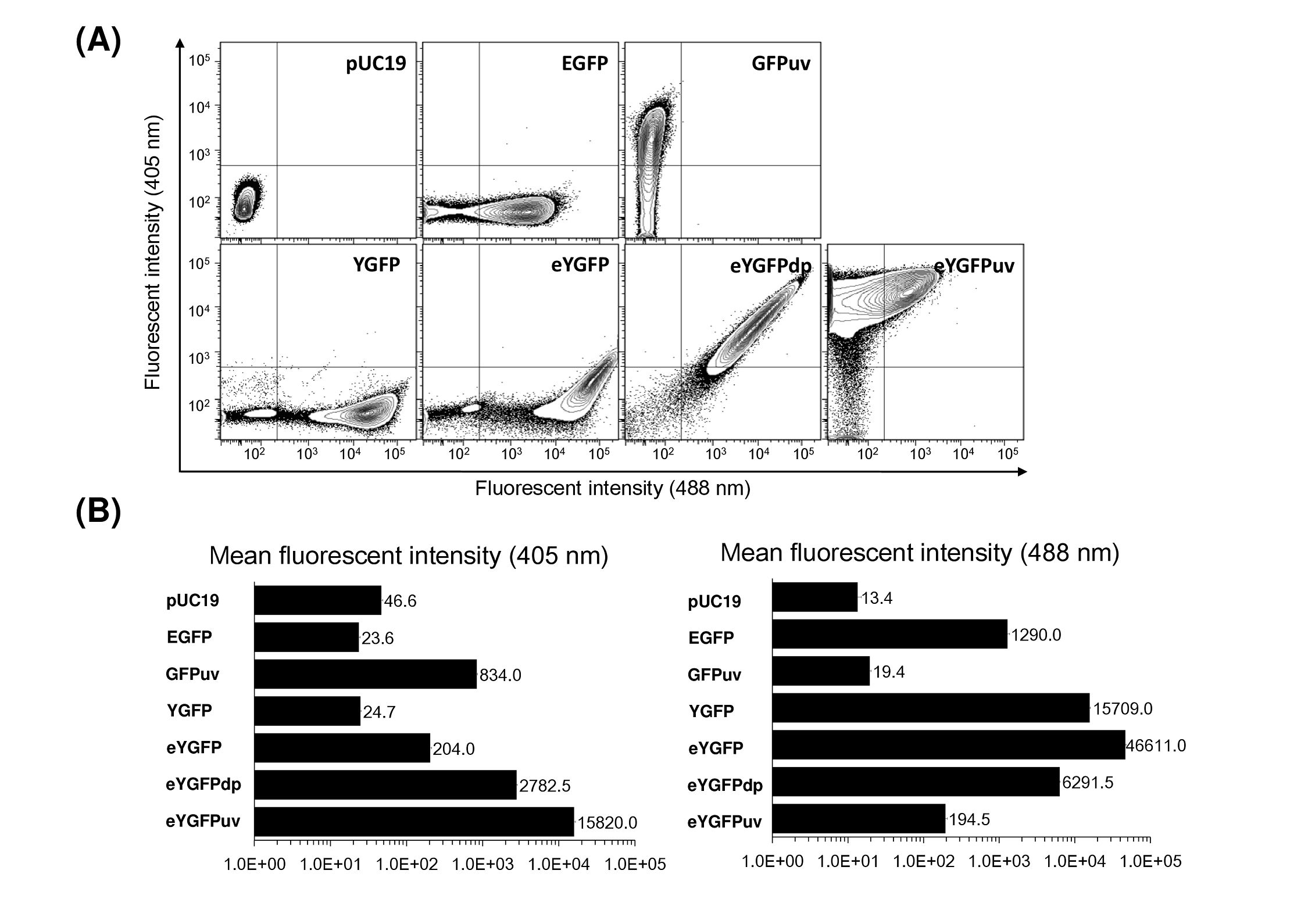

Supplement: S1 Fig — A His-tag was fused to the N-terminus of each FP. Flow cytometry was carried out using the same settings as described in Fig 5. The average fluorescence intensity of eYGFP expressing cells in the 488 nm channel was 3-fold greater than that of YGFP expressing cells. The mean fluorescence intensity of eYGFPuv expressing cells in the 405 nm channel was 20-fold greater than that of GFPuv expressing cells. The values represent the average of three independent experiments. (TIFF) [file pone.0181186.s001.tiff]

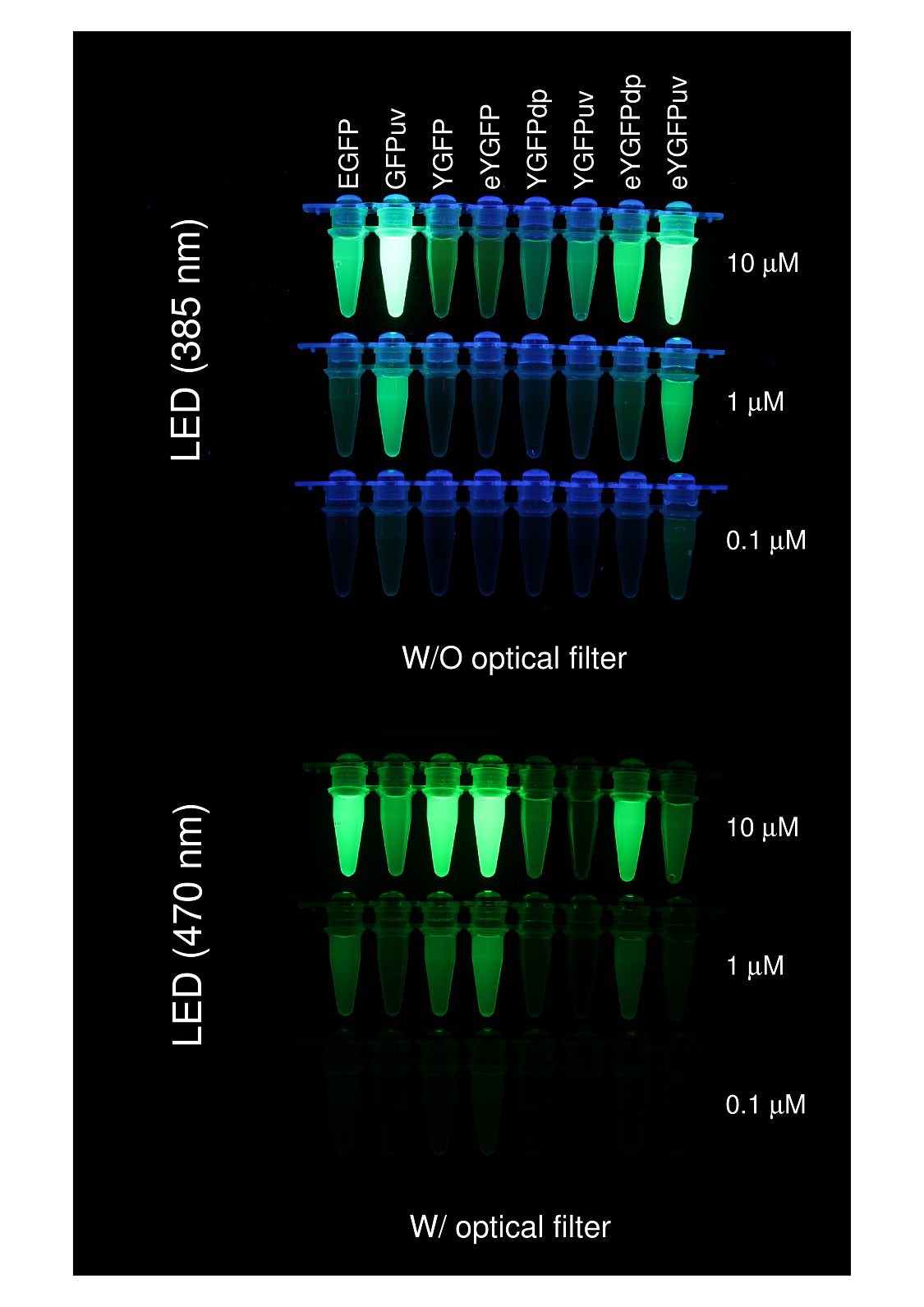

Supplement: S2 Fig — Photograph of bacterial colonies of each FP construct at indicated concentrations were taken under ultraviolet LED light (model LED-UV385P, OptoCode, Tokyo, Japan) without filters or blue LED light (model LED470-3WOF, OptoCode) with a yellow optical filter (SC52, Fujifilm, Tokyo, Japan) by using a Canon EOS Kiss Digital X7i camera (Canon, Tokyo, Japan). Image acquisition conditions: F5.6, ISO200, 1 s exposure, focal length 55 mm. Representative data from more than three independent experiments are shown. (TIFF) [file pone.0181186.s002.tiff]

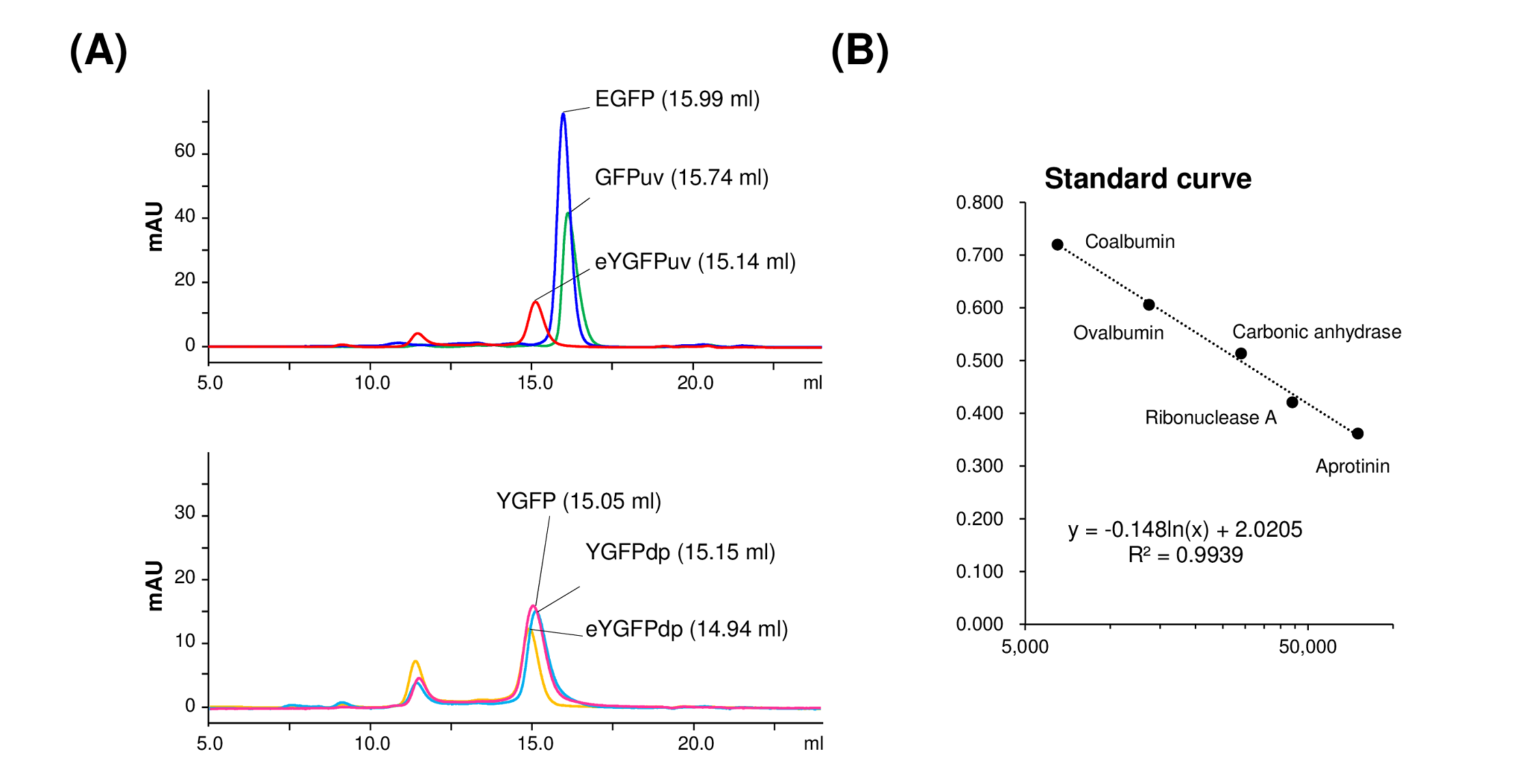

Supplement: S3 Fig — (A) 50 μg of each FP in PBS (20 mM, pH = 7.4) was loaded onto a Superdex 200 Increase 10/300 GL column. Expected elution volumes are indicated. (B) Standard curve for calibration of S200 size exclusion column. (TIFF) [file pone.0181186.s003.tiff]

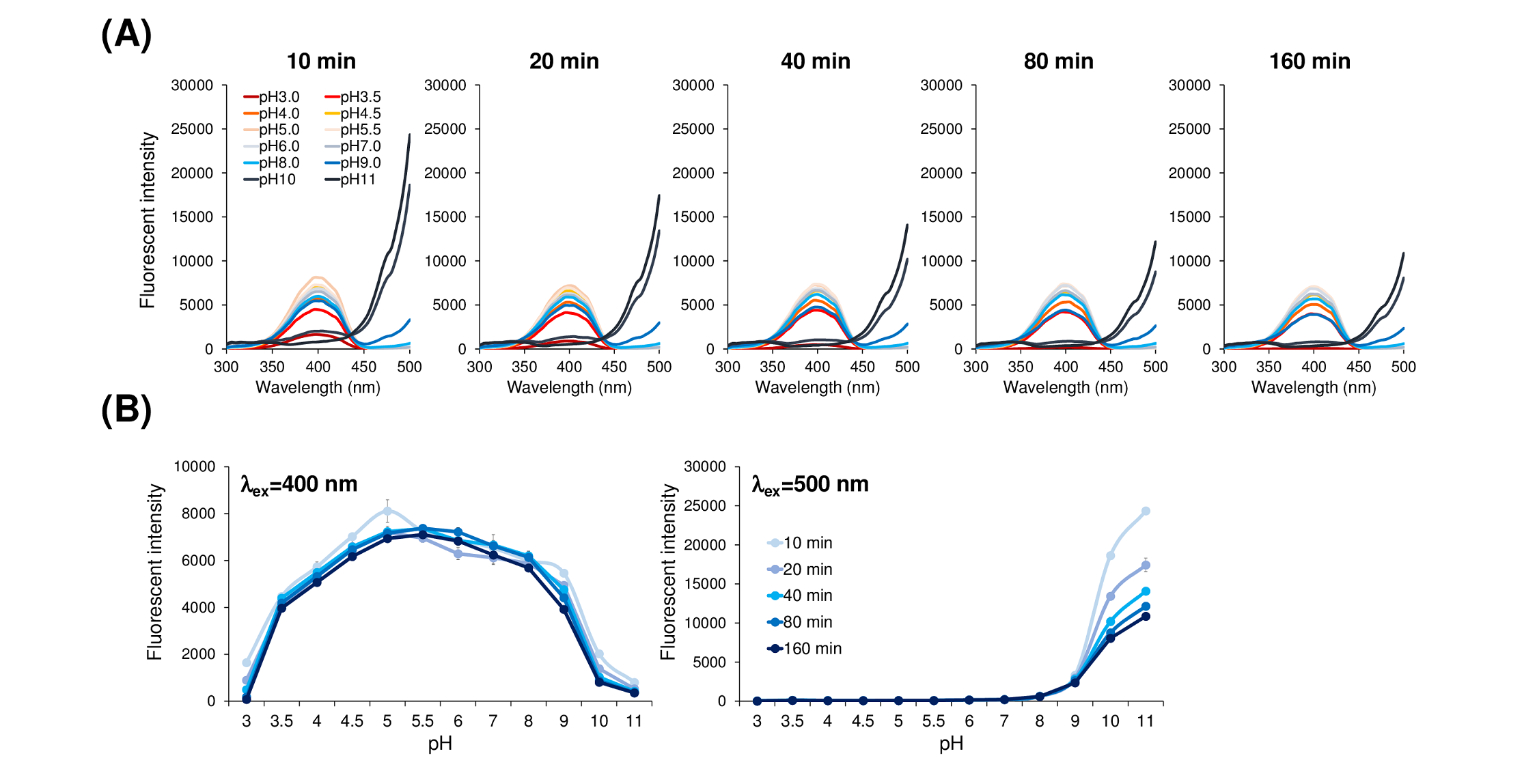

Supplement: S4 Fig — pH- and time-dependent changes of excitation spectra of eYGFPuv with emission maxima at 508 nm (upper panel) and plot of fluorescence versus pH at indicated excitation wavelength and peak emission wavelength (lower panel). In total, 20 μM of each fluorescent protein in PBS was diluted 10-folf with the indicated pH solutions and incubated at 25°C for the specified time durations. All experiments in this figure were repeated three times with each data point measured in triplicate; representative data are shown. (TIFF) [file pone.0181186.s004.tiff]

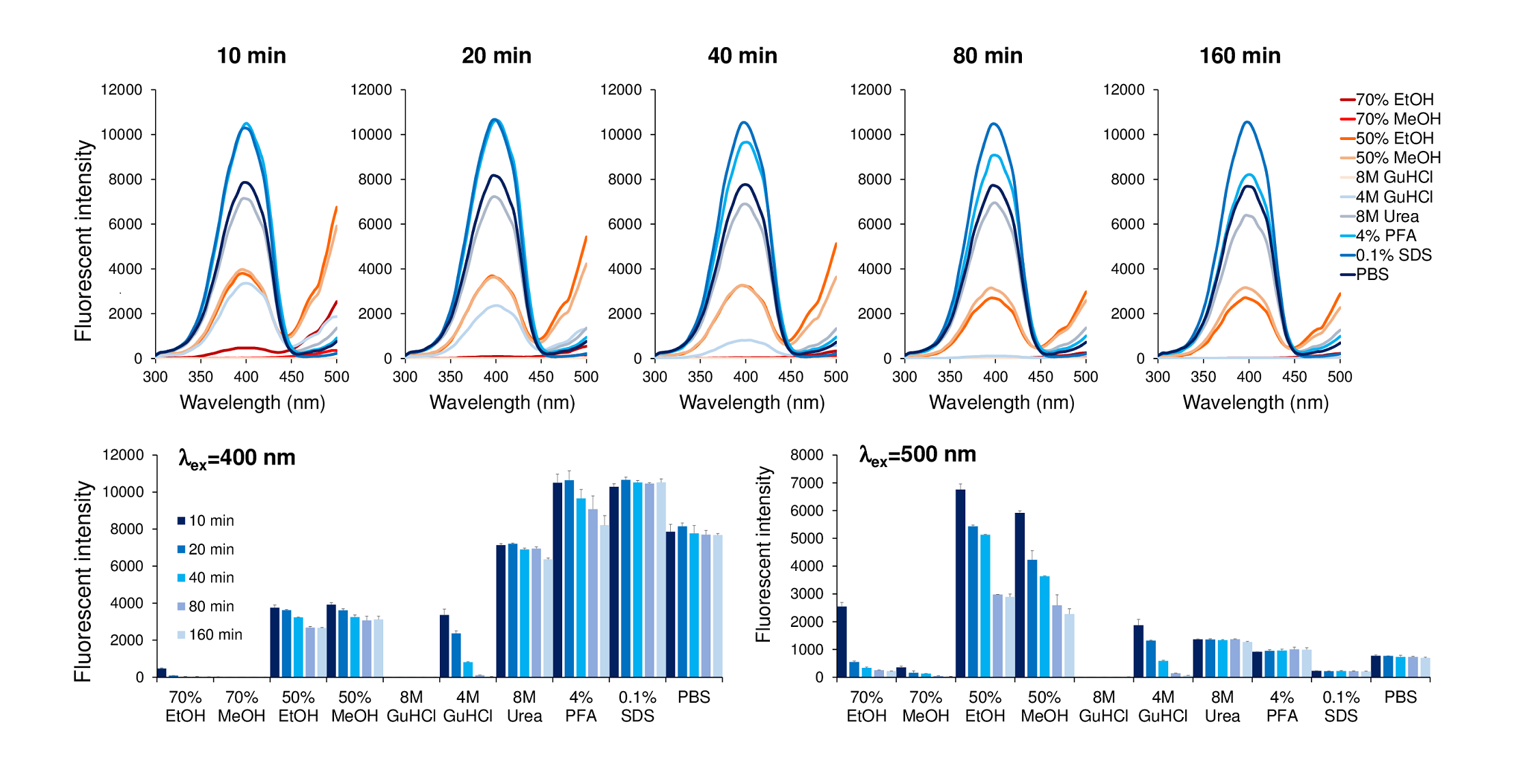

Supplement: S5 Fig — Fluorescence excitation spectra of eYGFPuv with emission maxima at 508 nm (upper panel) and fluorescence at indicated excitation wavelength and peak emission wavelength (lower panel) were normalized to a control sample diluted in PBS. In total, 20 μM of each fluorescent protein in PBS was diluted 10-fold with the indicated reagents and incubated at 25°C for the specified time durations. All experiments in this figure were repeated three times with each data point measured in triplicate; representative data are shown. (TIFF) [file pone.0181186.s005.tiff]

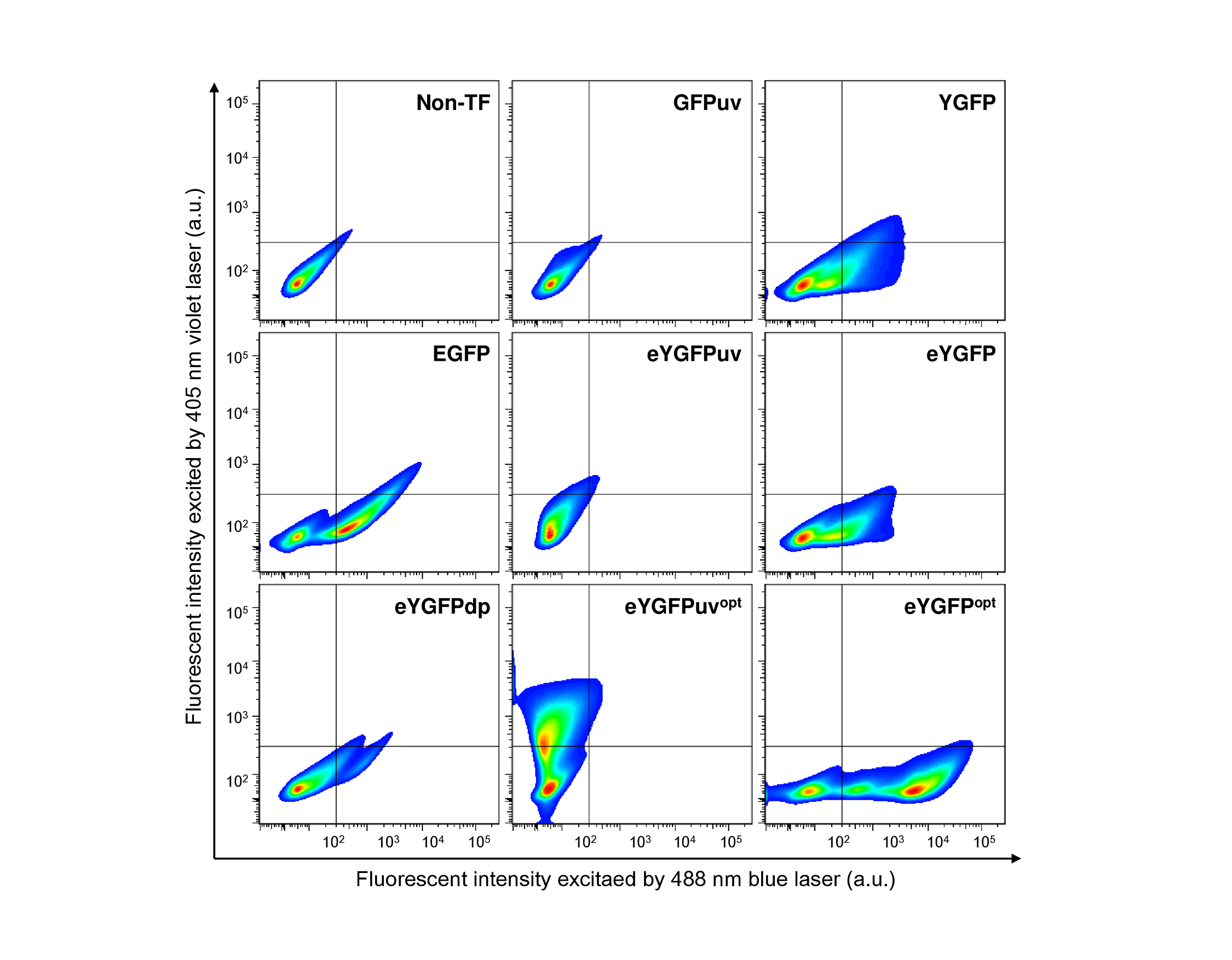

Supplement: S6 Fig — HCT116 cells expressing various FPs were prepared as described in Materials and Methods. FACS analysis was carried out using the same settings as described in Fig 6. Data are representative of two independent experiments. (TIFF) [file pone.0181186.s006.tiff]

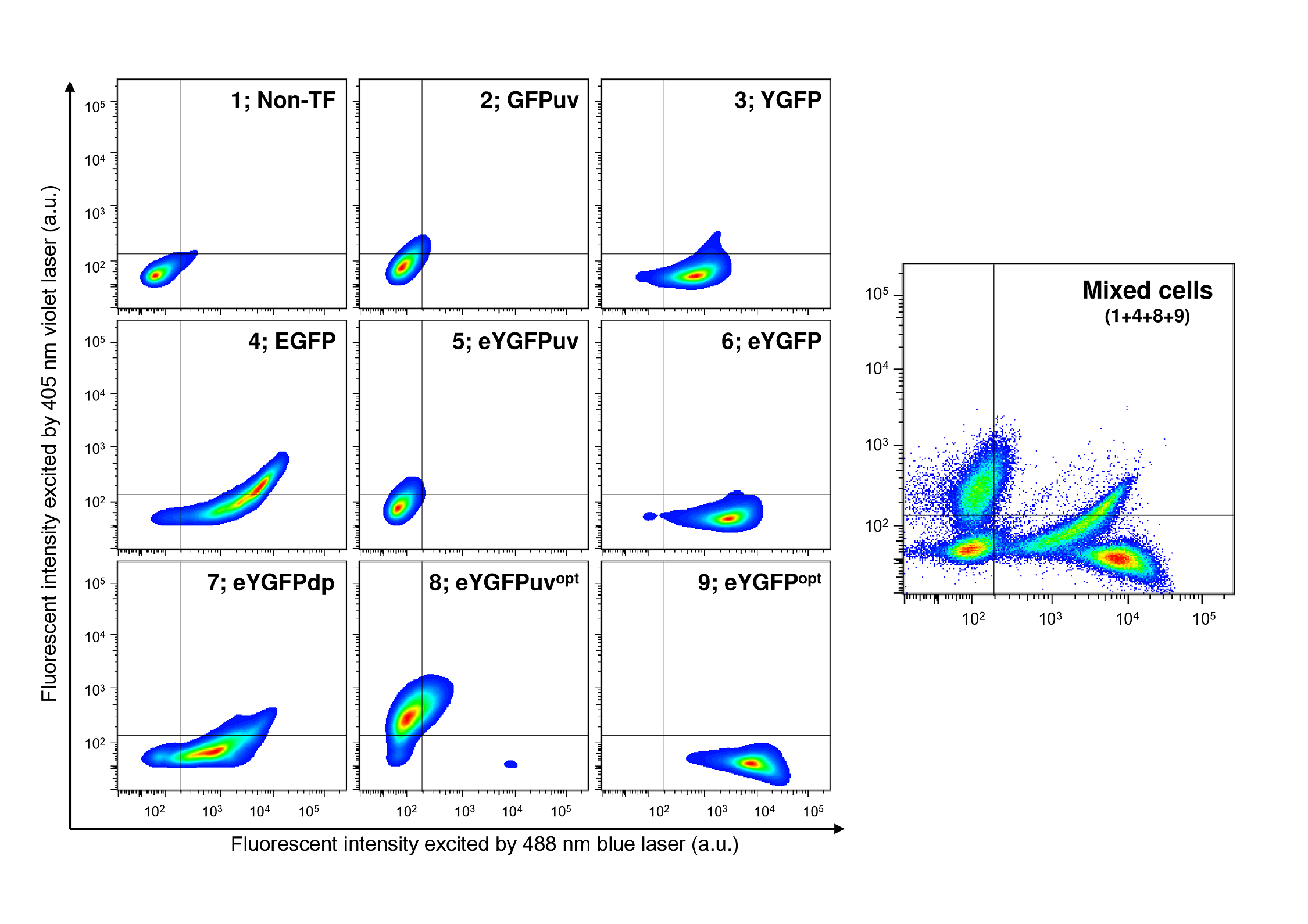

Supplement: S7 Fig — Preparation of MCF7 cells expressing various FPs and FACS analysis of sorted cells were as described for HCT116 cells. Mean fluorescence of purified cells is shown in Supplemental Table 1. Data are representative of three independent experiments. (TIFF) [file pone.0181186.s007.tiff]

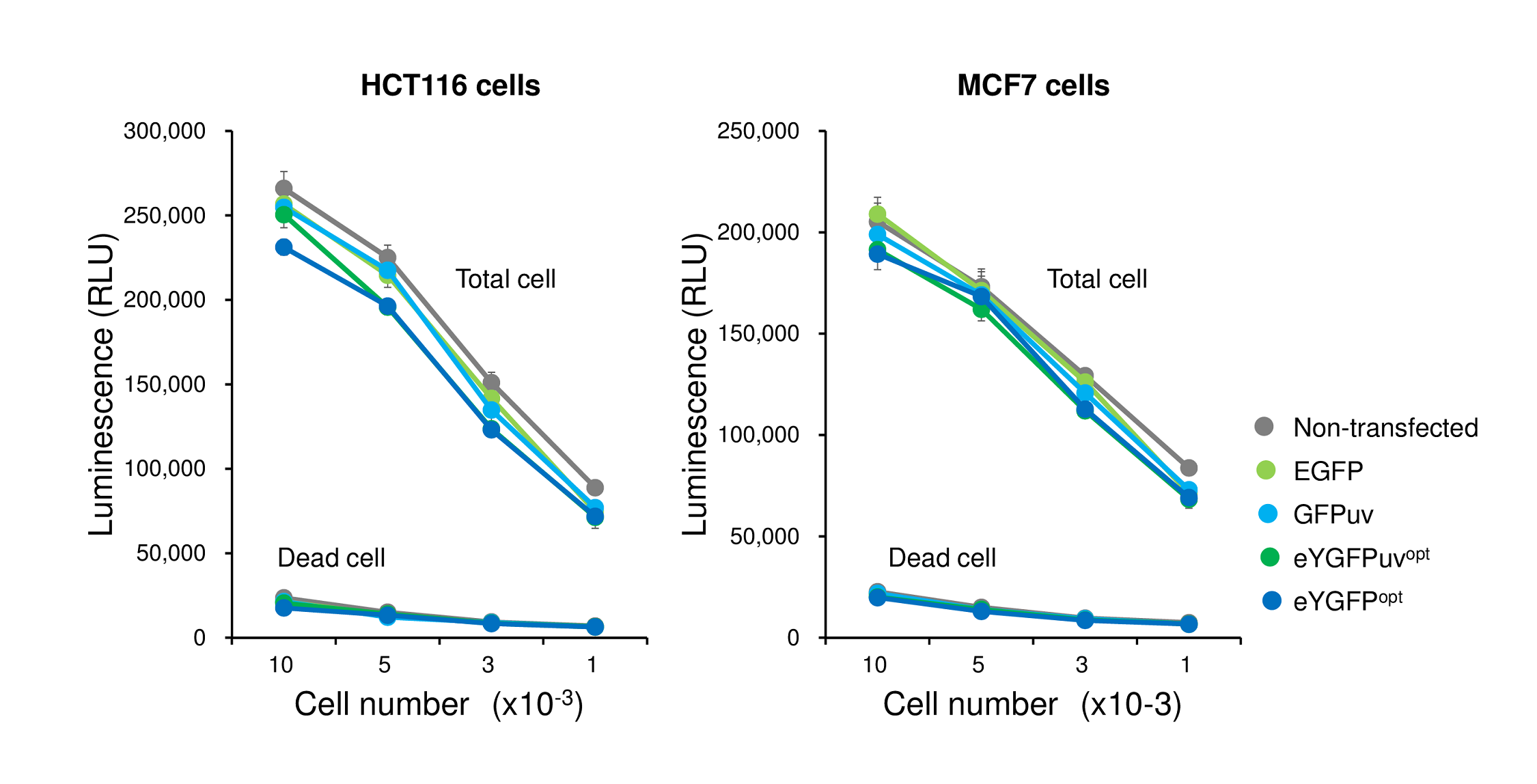

Supplement: S8 Fig — Cells were prepared as described in Materials and Methods. The experiment was repeated three times with each data point measured in triplicate; representative data are shown. (TIFF) [file pone.0181186.s008.tiff]

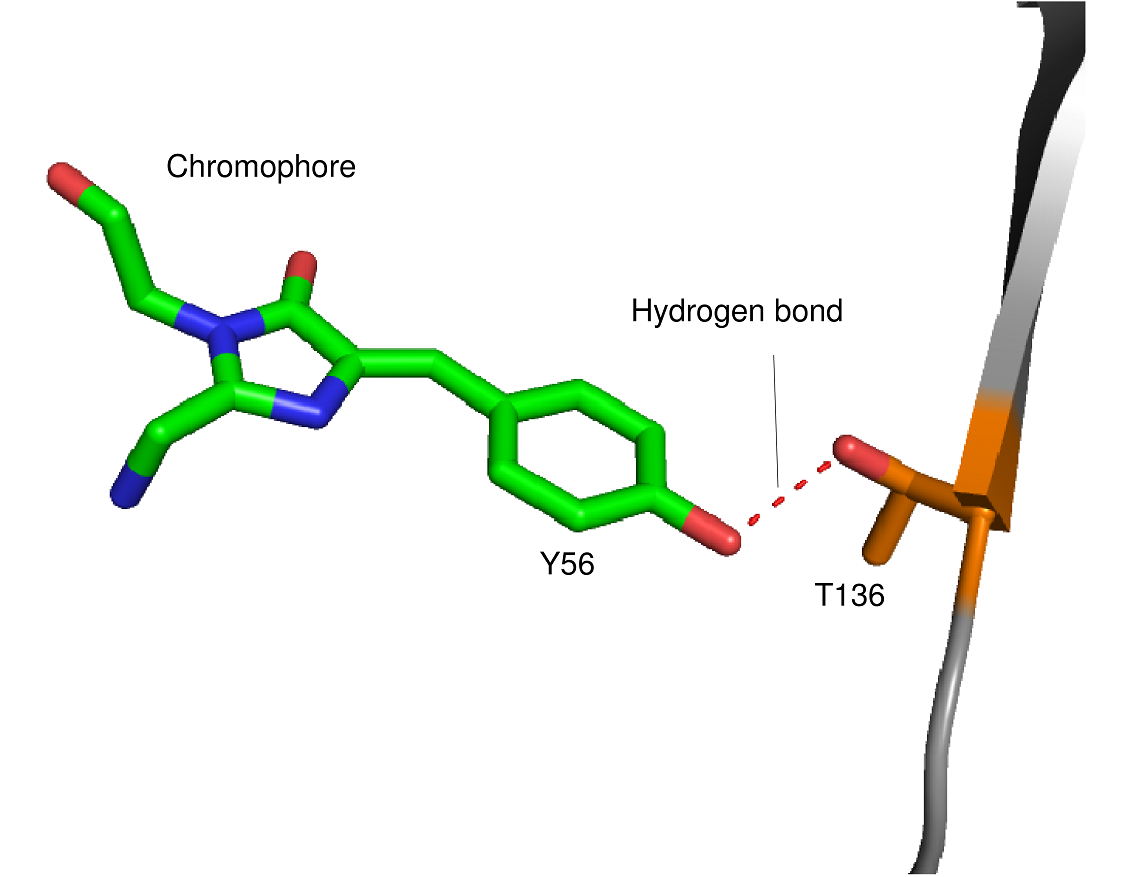

Supplement: S9 Fig — The structure model was drawn using PYMOL software as described in Fig 1. (TIFF) [file pone.0181186.s009.tiff]

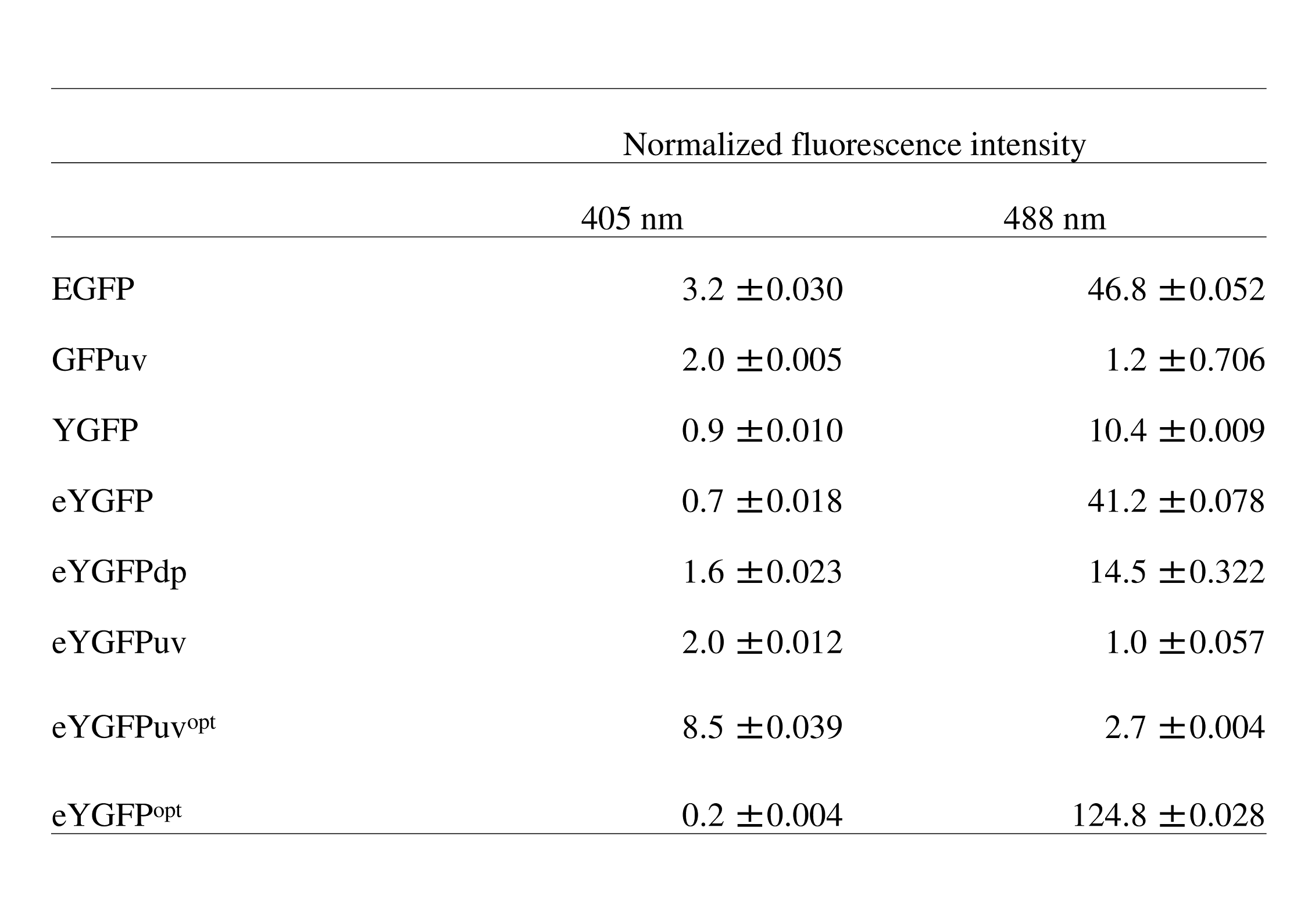

Supplement: S1 Table — The averages of fluorescence intensities for each fluorescent channel were normalized to those of non-transfected cells. The values represent the average of three independent experiments. (TIFF) [file pone.0181186.s010.tiff]

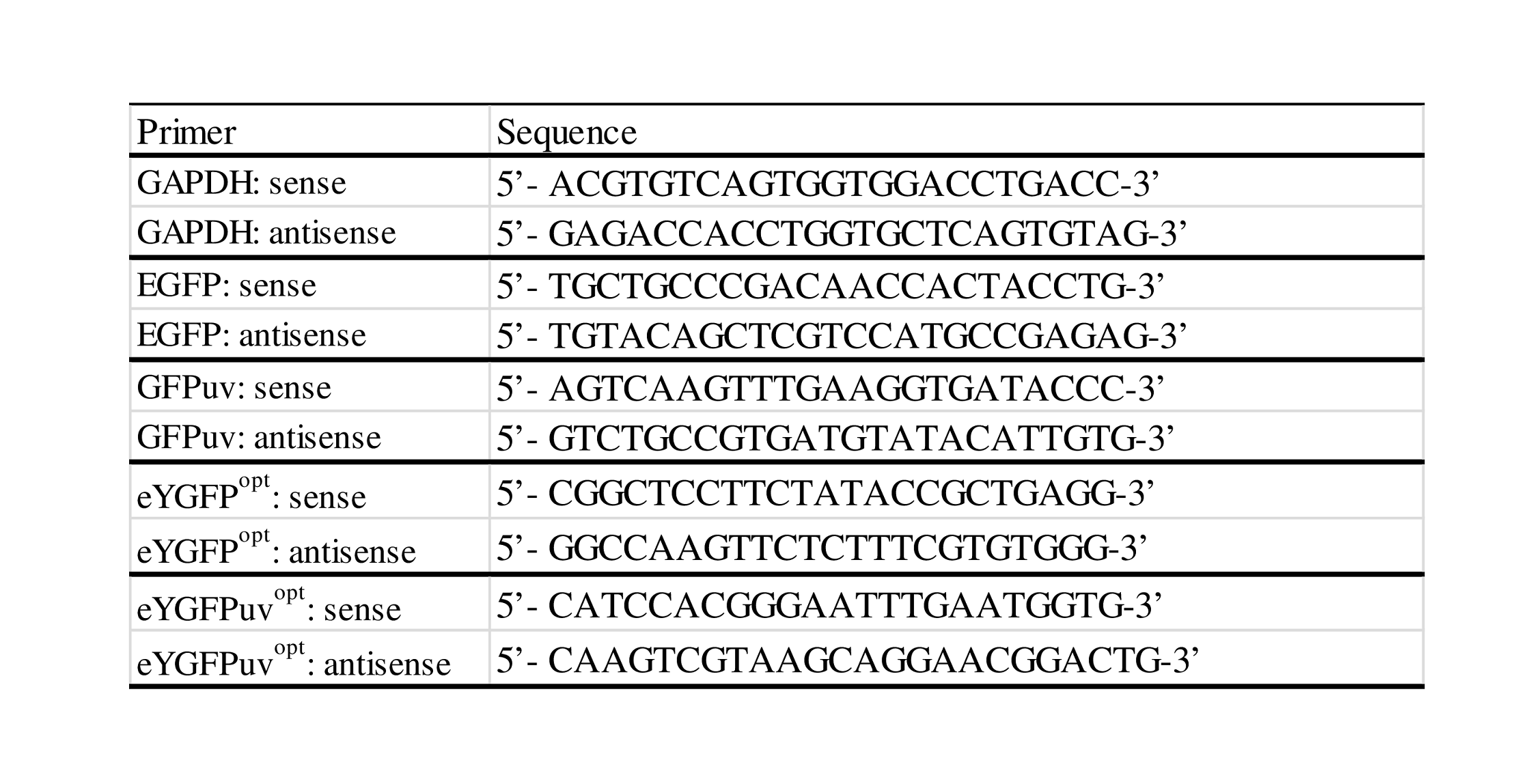

Supplement: S2 Table — (TIFF) [file pone.0181186.s011.tiff]
